# A multi-task segFormer framework for lesion segmentation and cerebral palsy classification based on multi-modal MRI in infant with periventricular white matter injury

**DOI:** 10.3389/fnins.2026.1870625

**Published:** 2026-07-02

**Authors:** Tingting Huang, Yitong Bian, Liang Wu, Fei Wang, Jian Yang

**Affiliations:** 1 Department of Radiology, The First Affiliated Hospital of Xi'an Jiaotong University, Xi'an, China; 2 Department of MRI, The First Affiliated Hospital of Henan University of Chinese Medicine, Zhengzhou, China; 3 Xi'an Key Laboratory of Medical Computational Imaging, Xi'an, China; 4 Shaanxi Engineering Research Center of Computational Imaging and Medical Intelligence, Xi'an, China; 5 Department of Children's Rehabilitation, Shaanxi Rehabilitation Hospital, Xi'an, China; 6 Department of Neurosurgery, Zhengzhou Second Hospital, Zhengzhou, China

**Keywords:** cerebral palsy, deep learning, MRI, multi-task classification, periventricular white matter injury

## Abstract

**Objective:**

This study aimed to develop a multi-task framework for detection of five MRI predictors of CP and intelligent recognition of CP based on multi-modal MRI in infant with PVWMI.

**Methods:**

We present MMSeg-CP, a multi-task framework for joint anatomical target region segmentation, lesion of target region segmentation, and CP classification from registered T1-weighted imaging (T1WI) and T2-weighted imaging (T2WI). MMSeg-CP adopts a SegFormer-based hierarchical transformer encoder and a lightweight all-MLP decoder, followed by lesion prediction and AttentionPool2d-based classification heads for infant neuroimaging characteristics, and performance was evaluated through five-fold cross-validation against nine comparative architectures using overlap, boundary, and classification metrics.

**Results:**

The study included 122 PVWMI infants (90 PVWMI with CP and 32 PVWMI with non-CP) and 121 infants with normal MRI. In five-fold cross-validation, the model achieved mean Dice values of 0.79 for target regions and 0.41 for lesions of target region, along with 0.95 slice-level accuracy and 0.88 subject-level accuracy. Compared with nine representative baseline models, MMSeg-CP provided the best overall balance between overlap accuracy, boundary precision, specificity, and sensitivity.

**Conclusion:**

MMSeg-CP enables joint detection of five MRI predictors of CP and intelligent CP recognition, supporting its potential as a clinical decision-support tool for early CP screening.

## 1 Introduction

Cerebral palsy (CP) is a heterogeneous group of permanent disorders of movement and posture resulting from non-progressive disturbances in the developing fetal or infant brain (Rosenbaum et al., 2007). It is the most common cause of long-term physical disability in childhood, with a global prevalence of approximately 1.5–3 per 1000 live births (McIntyre et al., 2022). The infant brain exhibits high neuroplasticity during the first 2 years of life (Gilmore et al., 2018; Yu et al., 2023). Therefore, early diagnosis of CP before the age of 2 facilitates early intervention and treatment, which significantly improves clinical outcomes for affected children (Morgan et al., 2021).

Periventricular white matter injury (PVWMI) is the most frequent MRI finding in infants with CP (56%) and is the main cause of CP (Himmelmann et al., 2017). Previous study based on conventional MRI has revealed that injuries involving the centrum semiovale, posterior limb of the internal capsule (PLIC), cerebral peduncle, thalamus, and lentiform nucleus are closely associated with CP in infants with PVWMI aged 6–24 months (Huang et al., 2025). However, MRI interpretation in this age group is difficult. The immature myelination status in infants younger than 2 years yields poor gray-white matter differentiation on MRI, and the heterogeneity of PVWMI lesions in morphology and signal intensity increases the difficulty and variability of manual interpretation. Subjective assessment of these imaging signs depends on radiological experience and presents significant challenges for non-specialists. Therefore, automated detection of these five MRI features coupled with intelligent recognition of CP would facilitate early screening and clinical applicability.

Deep learning has substantially advanced medical image analysis and demonstrated strong potential for multimodal lesion segmentation and disease classification (Akkus et al., 2017; Menze et al., 2015; Qian et al., 2024; Zhang et al., 2025). In particular, multimodal fusion networks, CNN-transformer hybrids, and multi-task architectures have improved performance in adult brain tumor imaging and related segmentation tasks by combining complementary contrasts with multi-scale contextual information (Aamir et al., 2022; Akkus et al., 2017; Luo et al., 2025; Menze et al., 2015; Shi et al., 2023; Xiang et al., 2022; Yamanakkanavar et al., 2020; Yang et al., 2023; Zhang et al., 2015; Zhao et al., 2024; Zhou et al., 2020; Zhang et al., 2024). More recently, these architectures have been increasingly applied to neonatal and pediatric neuroimaging, yielding significant improvements in neonatal brain segmentation, white matter hyperintensity detection (Hassan et al., 2025; Li et al., 2019). Within CP research specifically, multi-sequence MRI combined with 3D U-Net architectures has been used to segment CP-susceptible brain regions and spastic hemiplegic lesions (Hassan et al., 2024; Simarro et al., 2025), providing important methodological foundations for subsequent investigations. Nevertheless, in infants with PVWMI, lesions are typically much smaller and less conspicuous, whereas the available cohorts remain relatively modest. Moreover, clinically meaningful classification depends not merely on the presence of abnormal signal but also on its specific anatomical location. Therefore, the direct transfer of generic multimodal segmentation architectures is unlikely to be optimal for this task.

Therefore, we present MMSeg-CP, a multi-task framework that jointly performs target-region segmentation on T1-weighted imaging (T1WI), lesion segmentation on registered T2-weighted imaging (T2WI), and slice-level CP classification within a single optimization pipeline. This framework aims to enable joint detection of five MRI predictors of CP and intelligent CP recognition for early CP screening.

## 2 Materials and methods

### 2.1 Study cohort

This retrospective study included 122 PVWMI infants (mean age: 15 ± 4.3 months, range: 6–24 months) and 121 infants with normal MRI (mean age: 15 ± 5.3 months, range: 6–24 months). PVWMI was defined as bilateral signal abnormality or volume loss in the periventricular or deep white matter, with or without ventricular dilation, ventricular scalloping, or cystic change (Marefi et al., 2022). Exclusion criteria for the PVWMI cohort included inherited metabolic disease, congenital malformation, and motion artifacts. Among the PVWMI infants, 90 developed CP (15 ± 5.6 months) and 32 did not (14 ± 5.2 months). All PVWMI infants were followed up to 5 years of age, and CP diagnosis was made by a multidisciplinary team of pediatric neurologists and pediatric rehabilitation specialists (with 5–20 years of experience) in accordance with the international guidelines (Rosenbaum et al., 2007). The normal MRI cohort comprised infants who underwent MRI for non-neurological indications. All had normal neurological examinations and normal MRI. Exclusion criteria included nervous system infection, developmental delay, hypertonia, congenital heart disease, or absent follow-up neurological evaluation. This study was approved by the institutional review board (No. 2023HL-006-01) and the requirement for written informed consent was waived because of the retrospective design and the use of anonymized imaging data.

### 2.2 MRI acquisition and image preprocessing

MRI examinations were acquired on 3.0 T and 1.5 T scanners and included axial T1WI and axial T2WI sequences. Details of the MRI equipment and imaging protocols are provided in Supplementary Table S1. Slice thickness ranged from 5 to 7 mm, and the total number of slices per subject was 14, 15, or 18 (Supplementary Table S2). The through-plane resolution was relatively coarse, so we adopted a 2D slice-wise modeling strategy rather than volumetric modeling. Most CP positive subjects had only 3–5 lesion containing slices (Supplementary Table S3).

A radiologist with 14 years of pediatric neuroradiology experience manually delineated five target regions on T1WI (centrum semiovale, PLIC, cerebral peduncle, thalamus, and lentiform nucleus) and identified lesions in those regions on T2WI using ITK-SNAP (Supplementary Figure 1). Lesion delineation on T2WI was performed on original, unregistered slices with simultaneous visual reference to the corresponding T1WI slice and its predrawn target region masks, ensuring anatomically constrained lesion localization. The normal MRI cohort served as negative controls for CP classification and provided normal anatomical references for target-region segmentation. During training, normal cases contributed T1WI anatomical masks, all-zero lesion masks on T2WI, and non-CP slice labels.

To validate the consistency of these manual annotations, a second pediatric neuroradiologist (8 years of experience) independently annotated a random stratified sample of 40 PVWMI cases blinded to the original annotations. Inter-rater agreement was quantified by the Dice coefficient for segmentation masks and Cohen's kappa for slice-level CP classification.

All T1WI, T2WI, and mask slices were resampled to 512 × 512. Bilinear interpolation was applied to MRI intensities, and nearest-neighbor interpolation was used for segmentation masks to preserve label integrity. To ensure spatial consistency across modalities, each T2WI slice was aligned to its corresponding T1WI slice by 2D affine registration using the “antsRegistration” module in ANTs (Avants et al., 2011). The same identical geometric transformation was then applied to the lesion masks. After registration, image intensities were normalized to the range [0, 1] to stabilize optimization.

### 2.3 MMSeg-CP architecture

**Figure 1:**
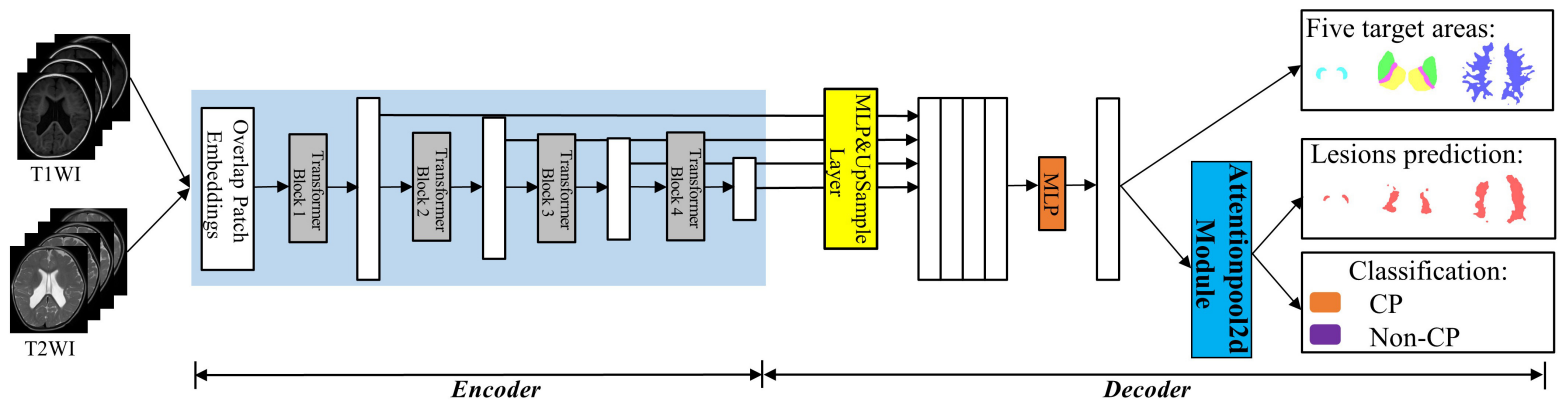
Overall architecture of MMSeg-CP for joint target region segmentation, target-region lesion segmentation, and slice-level CP classification.

The MMSeg-CP architecture was designed specifically to capture imaging and learning characteristics of PVWMI rather than to repurpose a generic segmentation template. It integrates a SegFormer-based hierarchical encoder with a lightweight decoder and multi-task heads to jointly process registered T1WI and T2WI. The encoder captures broad contextual dependencies and multi-scale representations, the decoder restores spatially detailed prediction maps with minimal added complexity, and multi-task supervision injects anatomical priors directly into representation learning. The overall architecture is illustrated in Figure 1.

#### 2.3.1 Hierarchical transformer encoder

MMSeg-CP adopts a SegFormer-based hierarchical transformer as its backbone because SegFormer combines efficient hierarchical self-attention with a lightweight decoder (Xie et al., 2021). Registered T1WI and T2WI slices are stacked as a multimodal input and fed to a shared encoder. Unlike the non-overlapping patch partitioning used in early vision transformers, the first stage uses overlapping patch embeddings to better preserve local continuity and boundary information, which is critical for delineating small lesions and narrow anatomical tracts.

**Figure 2:**
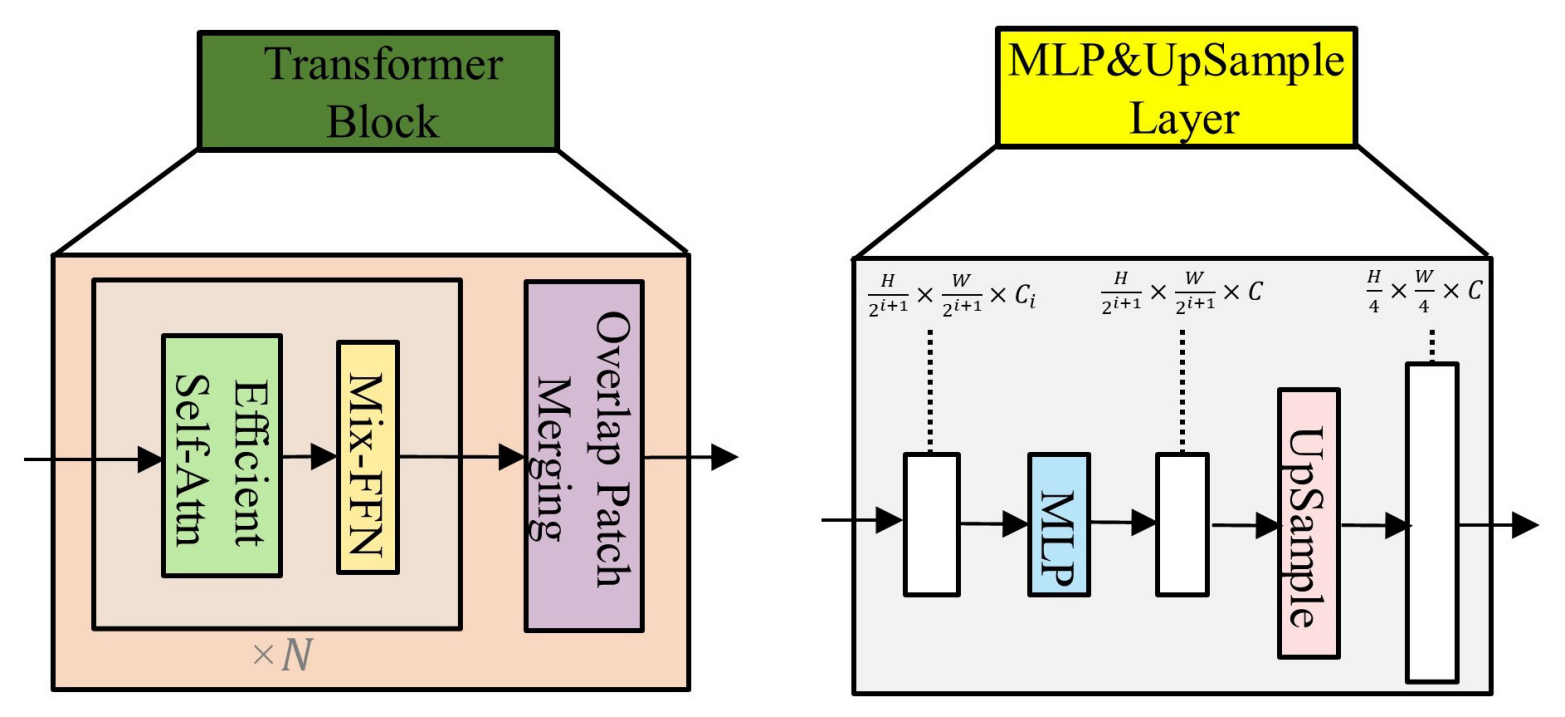
Internal structure of the transformer block, MLP and upsample layer in MMSeg-CP.

The encoder consists of four transformer stages that progressively reduce spatial resolution while increasing channel dimensionality. Each stage contains an efficient transformer block with self-attention and a Mix-FFN module, followed by overlapping patch merging. This yields multi-level features at 1/4, 1/8, 1/16, and 1/32 of the original image resolution, with shallow stages preserving more local structural detail and deeper stages capturing broader semantic context. The shared encoder promotes common multimodal features across all downstream tasks rather than isolated task-specific representations (Figure 2).

#### 2.3.2 Lightweight all-MLP decoder

The decoder of MMSeg-CP follows the lightweight all-MLP design principle of SegFormer (Xie et al., 2021). Multiscale features from the four encoder stages are first projected to a unified channel dimension by independent MLP layers. These feature maps were then upsampled to 1/4 of the input resolution and concatenated. A fusion MLP is subsequently applied to integrate information across scales, producing a compact representation that retains both detailed local structure and high-level semantic context. The decoder output is subsequently combined with an upsampling layer so that the final prediction maps match the input resolution (Figure 2).

After multi-modal encoding and decoding, the network branches into three task-specific outputs: (1) segmentation of five target regions, (2) segmentation of lesions of five target regions, and (3) slice-level classification into CP-related vs. non-CP slices. The target region head performs multiclass segmentation of the anatomically informative structures, whereas the lesion head performs binary delineation of abnormal signal on the lesion-sensitive representation. In parallel, an AttentionPool2d module aggregates decoder features into a compact global descriptor for classification. This branch was introduced because slice-level prediction should depend on more than a single local hotspot; it should summarize distributed evidence while remaining sensitive to the lesion bearing regions emphasized by the segmentation branches. Subject-level classification is derived from slice-level predictions; if any slice from one subject is predicted to contain CP-associated PVWMI lesions, the subject is categorized as CP-positive.

This multi-task formulation couples dense anatomical supervision with pathology-sensitive lesion detection and case-level diagnostic classification within a single optimization pipeline. The target-region branch constrains the representation space toward clinically meaningful structures, the lesion branch focuses on abnormal hyperintensity in T2WI, and the classification branch aggregates distributed evidence into subject-relevant disease descriptors. Joint optimization allows anatomical priors, lesion cues, and diagnostic classification to reinforce each other during training.

### 2.4 Loss functions

For the segmentation tasks, we use a combination of Dice loss and cross-entropy loss. To quantify segmentation overlap, we used the Dice coefficient, computed as D = (2 × |XnY|)/(|X| + |Y|), where X represents the predicted mask and Y represents the reference annotation. For lesion segmentation on T2WI images, the loss function is defined in Equation 1:


$$\begin{array}{l} L_{l,T2}\;=\;1\;-\;\frac{1}{2}\overset{2}{\underset{i=1}{\sum }}D_{i}+L_{l,T2}^{ce} \end{array}$$ (1)

where *D*_0_denotes the Dice score of the background region (without lesions), *D*_1_ denotes the Dice score of the lesion region, and${\;L}_{l,T2}^{ce}$is the pixel-wise cross-entropy loss. Furthermore, for the segmentation of the five target regions on T1-weighted images, the loss function is defined in Equation 2:


$$\begin{array}{l} L_{l,T1}\;=\;1\;-\;\frac{1}{m}\overset{m}{\underset{i=1}{\sum }}D_{i}+L_{l,T1}^{ce} \end{array}$$ (2)

where *D*_0_ denotes the Dice score of the background excluding the target regions, and *D*_*i*_(*i* = 1, 2, ..., 5) denotes the Dice score corresponding to each of the five target regions. For binary classification (CP vs. non-CP), we use the cross-entropy loss *L*_*cl*_. The final training objective is in Equation 3:


$$\begin{array}{l} L_{total}\;=\;{\alpha \;(L}_{l,T2}\;+\;L_{cl})\;+\;{(1-\alpha )L}_{l,T1} \end{array}$$ (3)

where α is a weighting coefficient determined through optimization.

### 2.5 Training and evaluation

All experiments were performed using five-fold cross-validation at the subject level. Ablation experiments were conducted on the first fold. Data augmentation consisted of horizontal flipping with probability 0.5, Gaussian blurring, and random rotation around the image center within −11° to +11°. Model optimization was performed using the Adam optimizer, with the initial learning rate set to 1.5 × 10^(−5)^ and an exponential decay rate of 0.9. End-to-end training was implemented in Python 3.8 and PyTorch 1.10 on Ubuntu 20.04 using an NVIDIA RTX 4090 GPU and CUDA 11.3. The average diagnosis time for one subject was approximately 51.7 ms.

Segmentation quality was evaluated by Dice coefficient and the 95th percentile Hausdorff distance (HD95). Classification performance was quantified by accuracy, specificity, and sensitivity at both slice and subject levels. For comparative experiments, MMSeg-CP was evaluated against nine representative multimodal AI models. The nine baseline models were selected to cover (i) disease relevance (SGMAN, DeepPWML for white matter lesions), (ii) diverse multimodal fusion strategies (F2Net, ACMINet, MMCA-Net, and MoSNet), and (iii) representative architectural paradigms (H-DenseFormer for CNN–Transformer hybrids, MFIB for edgesemantic fusion, and MFEFnet for multi-scale feature exploration). For the baseline models, we used the network architectures and hyperparameter settings reported in their original publications, with only the necessary input- and output-layer adaptations required to accommodate the present multimodal MRI inputs and segmentation/classification labels. No additional task-specific architectural redesign or exhaustive hyperparameter tuning was performed.

### 2.6 *Post-hoc* analyses

To investigate the relationship between lesion size and segmentation performance, a *post-hoc* stratification analysis was performed across the five cross-validation folds. Lesion size was quantified as the foreground-pixel count in the resampled reference mask (termed resampled lesion burden) rather than physical volume. Within each fold, lesion-containing slices were sorted by resampled lesion burden and divided into tertiles: small ( ≤ first tertile), medium (between first and second tertiles), and large (> second tertile). Fold-specific tertiles were used to avoid imposing arbitrary fixed cutoffs on the highly skewed lesion-size distribution. Additionally, an exploratory threshold scan was conducted within each fold to identify the lesion-burden threshold below which Dice decreased substantially.

A blinded clinician assessment was conducted to validate the clinical localization accuracy of predicted lesion masks. Two pediatric neuroradiologists (8 and 14 years of experience) independently reviewed predicted lesion masks from 49 randomly selected cases. Each reader was presented with the original T2WI slice overlaid with the predicted lesion mask, without access to the ground-truth annotation, and rated whether the mask correctly localized the lesion region (yes/no). Inter-rater agreement was quantified using Cohen's kappa, and the overall localization success rate was defined as the proportion of cases rated successful by both readers.

### 2.7 Subject-level aggregation analysis

To evaluate whether subject-level pooling could reduce false positives caused by isolated noisy slices, we additionally tested several aggregation strategies. Seven aggregation strategies were compared against the original any-positive-slice rule: (i) maximum probability pooling; (ii) mean probability pooling across all slices; (iii) noisy-or pooling [1 – Π(1 – p_i)], where p_i is the CP probability of the i-th slice); (iv–vi) top-k mean pooling of the k highest slice-level probabilities (*k* = 2, 3, 4); and (vii–ix) positive-slice-count thresholding (requiring ≥ k positive slices, *k* = 2, 3, 4).

## 3 Results

### 3.1 Model performance

Inter-rater reliability between two pediatric neuroradiologists for manual annotations showed a mean Dice of 0.78 ± 0.06 for target regions and 0.64 ± 0.13 for lesions of target regions (Supplementary Table S4), supporting the validity of the ground-truth annotations. In fivefold cross-validation,

MMSeg-CP achieved mean Dice values of 0.79 for the five target regions and 0.41 for the lesions of five target regions. Slice-level classification reached a mean accuracy of 0.95, while subject-level accuracy reached 0.88 with mean specificity and sensitivity of 0.88 and 0.90, respectively (Table 1).

**Table 1:** Fold-wise overall segmentation and classification performance of MMSeg-CP.

Fold	Target-region dice	Lesion dice	Slice accuracy	Subject accuracy	Subject specificity	Subject sensitivity
0	0.80	0.44	0.94	0.86	0.80	0.95
1	0.80	0.38	0.96	0.90	0.85	1.00
2	0.79	0.38	0.95	0.90	0.93	0.84
3	0.78	0.42	0.95	0.90	0.93	0.85
4	0.79	0.45	0.94	0.88	0.87	0.88
Mean	0.79	0.41	0.95	0.88	0.88	0.90

CP, cerebral palsy

Table 2 shows that segmentation accuracy differed across anatomical regions. The thalamus and lentiform nucleus achieved the highest mean Dice (0.89, 0.86), reflecting their clear structural boundaries, whereas the centrum semiovale was more challenging (0.74).

**Table 2:** Fold-wise dice scores for the five target regions.

Fold	Centrum semiovale	PLIC	Cerebral peduncle	Thalamus	Lentiform nucleus	Mean
0	0.74	0.74	0.74	0.90	0.86	0.80
1	0.78	0.73	0.74	0.90	0.87	0.80
2	0.73	0.71	0.76	0.86	0.86	0.79
3	0.69	0.72	0.75	0.88	0.86	0.78
4	0.75	0.72	0.74	0.88	0.86	0.79
Mean	0.74	0.72	0.75	0.89	0.86	0.79

PLIC, posterior limb of the internal capsule

The boundary-based results in Table 3 are consistent with the overlap-based findings. Regions with clearer anatomical definition tended to show lower HD95 values, whereas the centrum semiovale and lesion masks exhibited larger distances because their contours were more irregular and their predicted boundaries were more vulnerable to small local deviations.

**Table 3:** Fold-wise HD95 values for the five target regions and lesions.

Fold	Centrum semiovale	PLIC	Cerebral peduncle	Thalamus	Lentiform nucleus	Mean	Lesion HD95
0	8.09	2.19	2.14	2.38	3.53	3.67	11.57
1	5.77	2.22	1.57	2.07	2.23	2.77	11.42
2	6.37	2.32	1.71	2.34	2.49	3.05	12.82
3	6.85	2.17	1.60	2.25	2.42	3.06	11.45
4	5.42	2.64	1.52	1.99	2.29	2.77	12.29
Mean	6.50	2.31	1.71	2.21	2.59	3.06	11.91

PLIC, posterior limb of the internal capsule; HD95: 95th percentile Hausdorff distance

**Figure 3:**
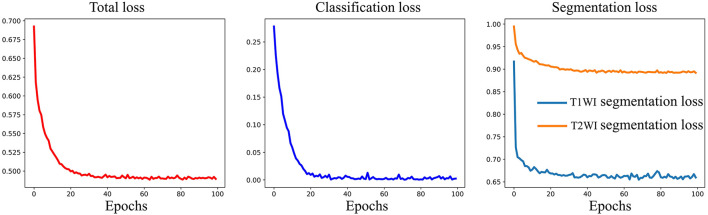
Training curves of the total, classification, lesion segmentation, and target-region segmentation losses.

Training dynamics are summarized in Figure 3. The joint objective decreased rapidly during early epochs and then gradually approached a stable plateau.

The classification loss converged after approximately 40 epochs, the target-region loss also declined in a smooth manner. The lesion-related component decreased more slowly. All three branches reached stable convergence without mutual interference.

### 3.2 Comparison with baseline methods

Tables 4–6 compare MMSeg-CP with nine representative baseline methods.

**Table 4:** Comparison of overall segmentation and classification results with nine baseline models (mean ± standard deviation).

Model	Target-region dice	Lesion dice	Slice accuracy	Subject accuracy	Subject specificity	Subject sensitivity
SGMAN (Wang et al., 2021)	0.73	-	0.80	-	-	-
DeepPWML(Ren et al., 2023)	0.76 ± 0.04^*^	0.37 ± 0.03	0.92 ± 0.01^**^	0.79 ± 0.06^*^	0.82 ± 0.06	0.75 ± 0.11^*^
F2Net (Yang et al., 2023)	0.66 ± 0.03^**^	0.39 ± 0.02	0.92 ± 0.02^*^	0.81 ± 0.03^**^	0.79 ± 0.08	0.85 ± 0.09
H-DenseFormer (Shi et al., 2023)	0.79 ± 0.01	0.39 ± 0.01	0.91 ± 0.01^*^	0.81 ± 0.05^**^	0.78 ± 0.06	0.85 ± 0.09
ACMINet (Zhuang et al., 2022)	0.78 ± 0.02	0.38 ± 0.03	0.93 ± 0.01^**^	0.81 ± 0.03^**^	0.80 ± 0.07	0.83 ± 0.12
MFIB (Zhu et al., 2023)	0.71 ± 0.05^*^	0.29 ± 0.05^**^	0.91 ± 0.02^**^	0.76 ± 0.07^*^	0.77 ± 0.12	0.76 ± 0.10^*^
MMCA-Net (Zhao et al., 2024)	0.76 ± 0.00^**^	0.34 ± 0.02^**^	0.93 ± 0.01^**^	0.78 ± 0.05^**^	0.75 ± 0.10^*^	0.82 ± 0.09
MoSNet (Xiang et al., 2022)	0.76 ± 0.02^*^	0.33 ± 0.07^*^	0.93 ± 0.01^**^	0.79 ± 0.02^**^	0.75 ± 0.06^*^	0.85 ± 0.04
MFEFnet (Zhang et al., 2025)	0.74 ± 0.03^**^	0.38 ± 0.02	0.93 ± 0.01^*^	0.79 ± 0.08^*^	0.77 ± 0.12	0.83 ± 0.07
MMSeg-CP (Ours)	**0.79** **±0.01**	**0.41** **±0.03**	**0.95** **±0.01**	**0.88** **±0.02**	**0.88** **±0.06**	**0.90** **±0.07**

CP: cerebral palsy. Bold values indicate the best performance achieved by the proposed MMSeg-CP model among all compared methods. ^*^indicates *P* < 0.05. ^*^^*^indicates *P* < 0.001.

MMSeg-CP achieved the highest mean target-region Dice (0.79 ± 0.01), the best overall lesion Dice (0.41 ± 0.03), the highest slice-level classification accuracy (0.95 ± 0.01), and the strongest subject-level sensitivity (0.90 ± 0.07) and specificity (0.88 ± 0.06) among the compared methods (Table 4).

Region-wise analysis showed that MMSeg-CP obtained the highest average Dice across the five target regions, particularly in the thalamus, lentiform nucleus, and cerebral peduncle (Table 5). The centrum semiovale remained the most difficult region for all methods, but MMSeg-CP produced the best average result. In terms of boundary precision, MMSeg-CP achieved the lowest average target-region HD95 and the lowest lesion HD95 (Table 6).

**Table 5:** Region-wise dice comparison with nine baseline models (mean ± standard deviation).

Model	Centrum semiovale	PLIC	Cerebral peduncle	Thalamus	Lentiform nucleus	Mean
SGMAN (Wang et al., 2021)	0.76	0.70	0.73	0.74	-	0.73
DeepPWML(Ren et al., 2023)	0.71 ± 0.06	0.68 ± 0.04^**^	0.72 ± 0.05	0.84 ± 0.04^**^	0.85 ± 0.02^*^	0.76 ± 0.04^*^
F2Net (Yang et al., 2023)	0.46 ± 0.09^**^	0.72 ± 0.01	0.35 ± 0.12^**^	0.89 ± 0.01	0.84 ± 0.02^*^	0.66 ± 0.03^**^
H-DenseFormer (Shi et al., 2023)	0.76 ± 0.01	0.71 ± 0.01	0.73 ± 0.01^*^	0.89 ± 0.00	0.86 ± 0.01	0.79 ± 0.01
ACMINet (Zhuang et al., 2022)	0.71 ± 0.04	0.71 ± 0.01^*^	0.72 ± 0.01^**^	0.88 ± 0.01	0.85 ± 0.02	0.78 ± 0.02
MFIB (Zhu et al., 2023)	0.70 ± 0.02	0.66 ± 0.05^*^	0.67 ± 0.03^**^	0.77 ± 0.09^*^	0.75 ± 0.08^*^	0.71 ± 0.05^*^
MMCA-Net (Zhao et al., 2024)	0.66 ± 0.04^*^	0.70 ± 0.01^**^	0.73 ± 0.01^*^	0.86 ± 0.03	0.84 ± 0.02	0.76 ± 0.00^**^
MoSNet (Xiang et al., 2022)	0.64 ± 0.07^*^	0.71 ± 0.02	0.73 ± 0.01^*^	0.86 ± 0.02	0.85 ± 0.03	0.76 ± 0.02^*^
MFEFnet (Zhang et al., 2025)	0.60 ± 0.07^*^	0.68 ± 0.05	0.73 ± 0.01	0.87 ± 0.01	0.81 ± 0.09	0.74 ± 0.03^**^
MMSeg-CP (Ours)	**0.74** **±0.03**	**0.72** **±0.01**	**0.75** **±0.01**	**0.89** **±0.01**	**0.86** **±0.01**	**0.79** **±0.01**

PLIC, posterior limb of the internal capsule. Bold values indicate the best performance achieved by the proposed MMSeg-CP model among all compared methods. ^*^indicates *P* < 0.05. ^*^^*^indicates *P* < 0.001.

**Table 6:** Region-wise HD95 comparison with nine baseline models (mean ± standard deviation).

Model	Centrum semiovale	PLIC	Cerebral peduncle	Thalamus	Lentiform nucleus	Mean	Lesion HD95
SGMAN (Wang et al., 2021)	-	-	-	-	-	-	-
DeepPWML(Ren et al., 2023)	7.92 ± 2.20	4.90 ± 2.32	2.55 ± 0.87	6.08 ± 2.90^*^	5.28 ± 1.56^*^	5.35 ± 1.58^*^	16.25 ± 1.54^**^
F2Net (Yang et al., 2023)	10.03 ± 1.08^**^	2.17 ± 0.27	2.46 ± 0.77	2.07 ± 0.26	2.98 ± 0.35	3.95 ± 0.36^*^	12.83 ± 1.44
H-DenseFormer (Shi et al., 2023)	7.51 ± 1.56	2.96 ± 0.71	2.22 ± 0.75	2.63 ± 0.55	3.14 ± 0.84	3.70 ± 0.76	14.95 ± 0.96^**^
ACMINet (Zhuang et al., 2022)	7.93 ± 1.01^*^	2.69 ± 0.68	2.02 ± 0.19	2.53 ± 0.40	2.98 ± 0.76	3.63 ± 0.45^*^	14.23 ± 1.16^**^
MFIB (Zhu et al., 2023)	15.18 ± 2.26^**^	4.78 ± 1.10^**^	5.04 ± 2.86^*^	4.62 ± 1.01^**^	5.96 ± 1.84^**^	7.12 ± 1.28^**^	16.34 ± 0.93^**^
MMCA-Net (Zhao et al., 2024)	12.15 ± 1.78^**^	3.10 ± 0.50^*^	1.90 ± 0.21	3.78 ± 1.58	3.78 ± 0.74^*^	4.94 ± 0.53^**^	17.34 ± 4.88^*^
MoSNet (Xiang et al., 2022)	14.13 ± 3.44^**^	2.65 ± 0.55	2.19 ± 0.72	2.41 ± 0.54	2.53 ± 0.60	4.78 ± 0.62^**^	14.64 ± 2.36^*^
MFEFnet (Zhang et al., 2025)	15.09 ± 3.35^**^	3.46 ± 0.49^**^	2.50 ± 0.17^**^	2.94 ± 0.42^*^	3.77 ± 1.27	5.56 ± 0.64^**^	15.13 ± 1.62^**^
MMSeg-CP (Ours)	**6.50** **±0.93**	**2.31** **±0.17**	**1.71** **±0.22**	**2.21** **±0.15**	**2.59** **±0.48**	**3.06** **±0.33**	**11.91** **±0.56**

PLIC, posterior limb of the internal capsule. Bold values indicate the best performance achieved by the proposed MMSeg-CP model among all compared methods. ^*^indicates *P* < 0.05. ^*^^*^indicates *P* < 0.001.

**Figure 4:**
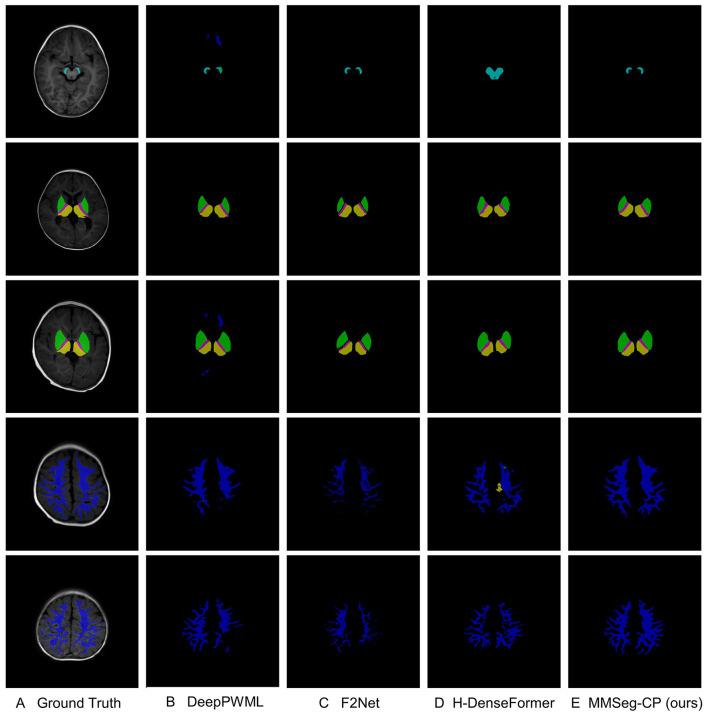
Qualitative comparison of target-region segmentation between MMSeg-CP and representative baseline methods. **(A)** Ground truth, **(B)** DeepPWML, **(C)** F2Net, **(D)** H-DenseFormer, **(E)** MMSeg-CP (ours).

**Figure 5:**
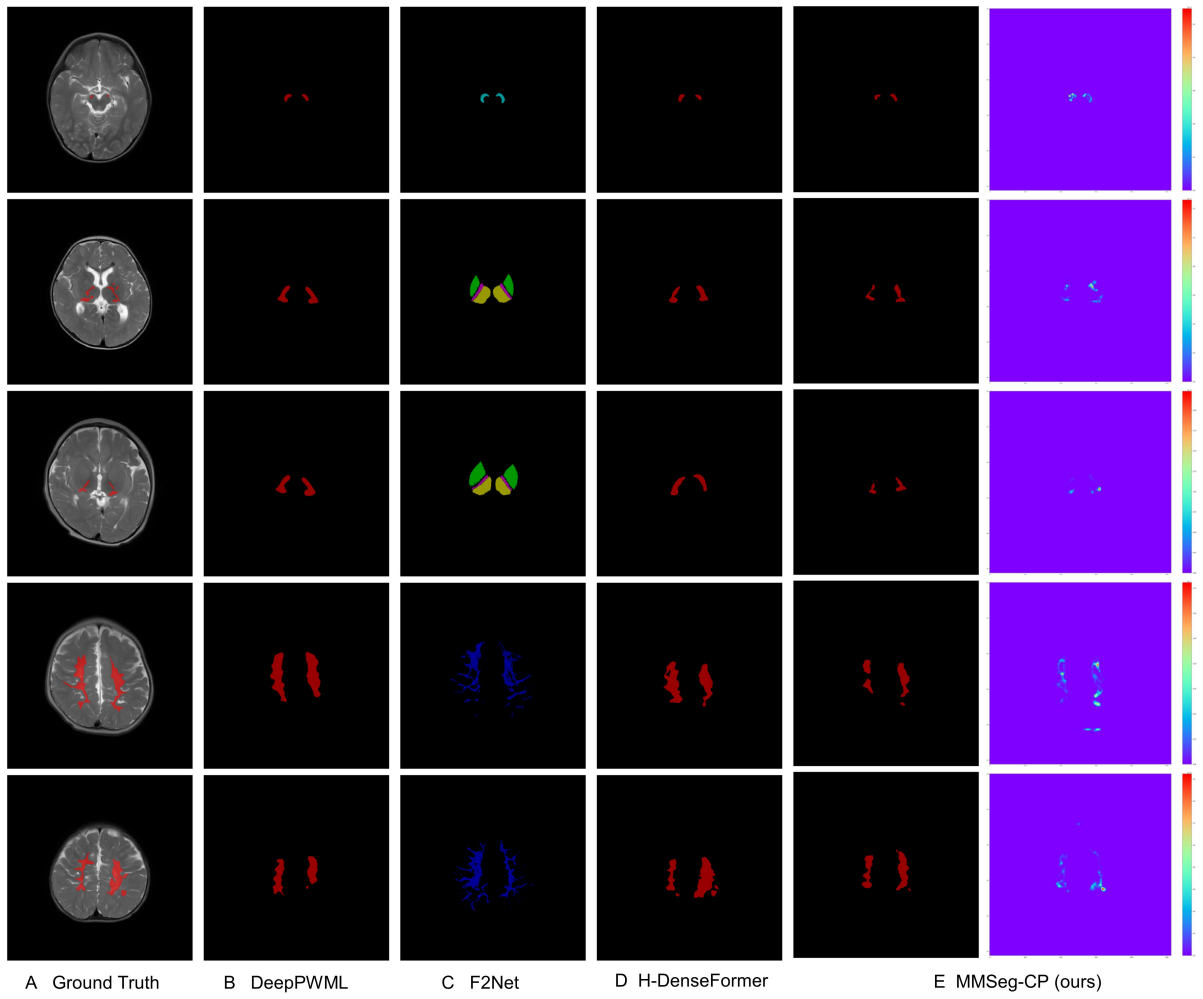
Qualitative comparison of lesion segmentation between MMSeg-CP and representative baseline methods. **(A)** Ground truth, **(B)** DeepPWML, **(C)** F2Net, **(D)** H-DenseFormer, **(E)** MMSeg-CP (ours).

**Figure 6:**
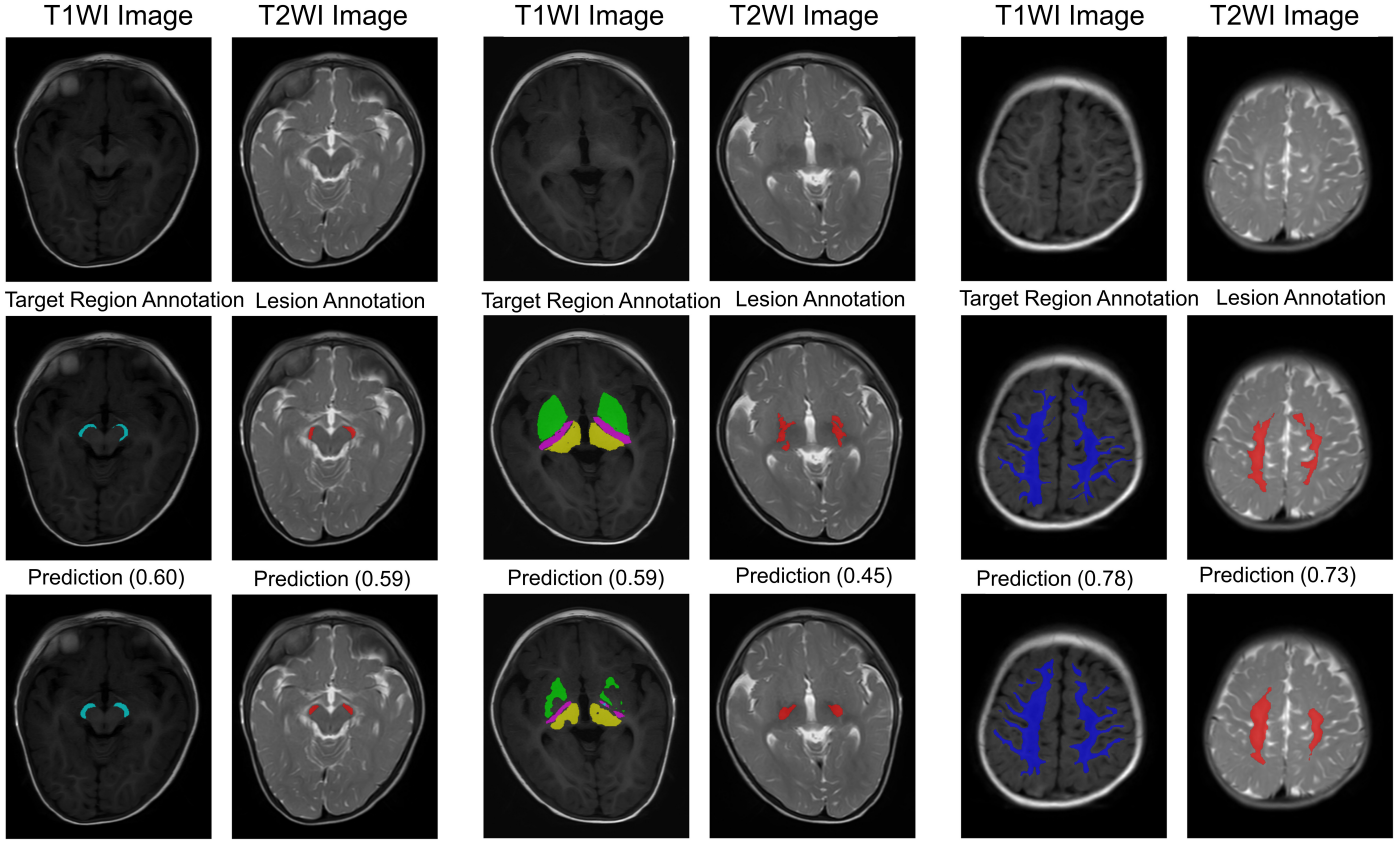
Representative MMSeg-CP predictions across varying lesion burdens and anatomical appearances.

**Figure 7:**
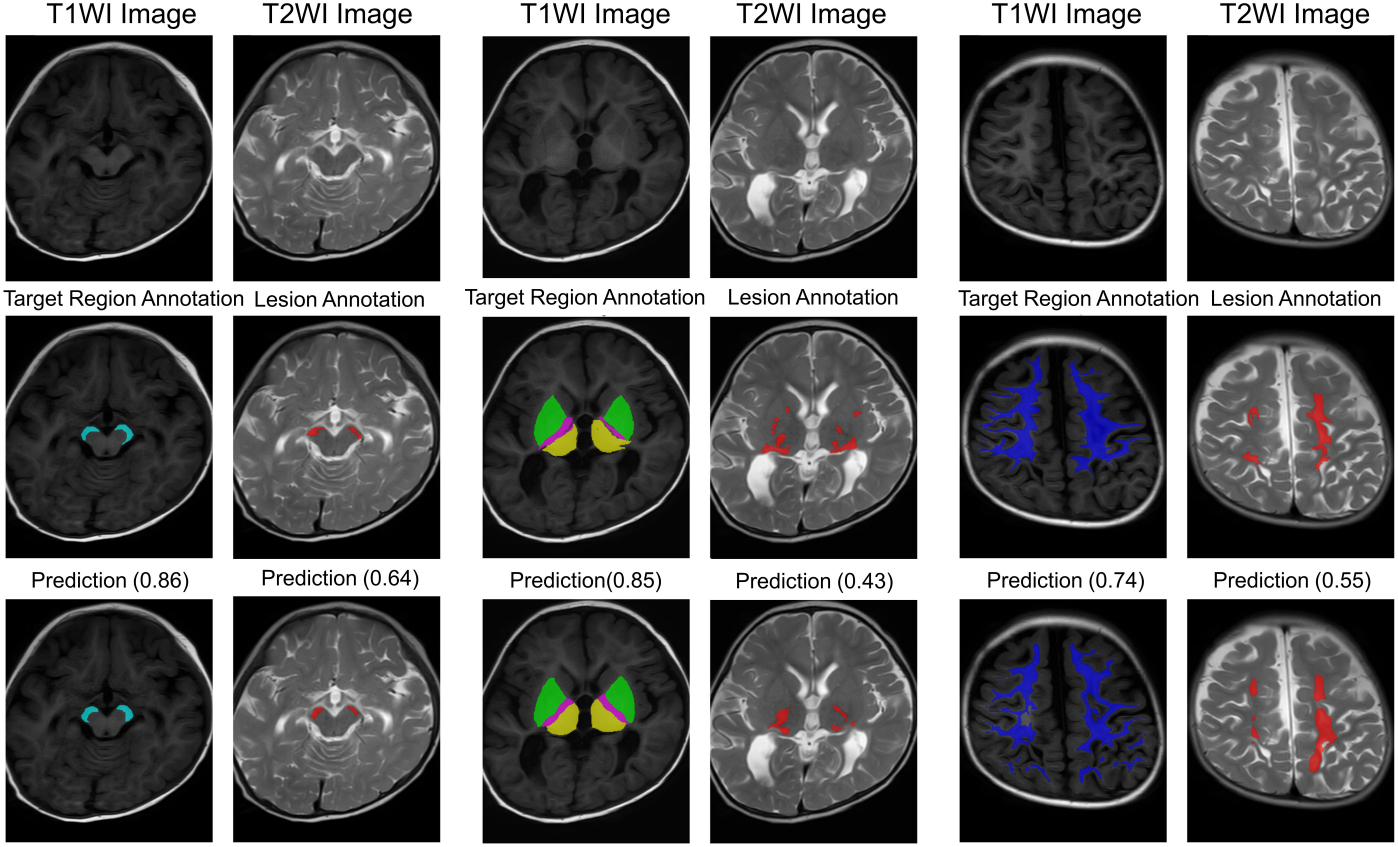
Representative MMSeg-CP predictions showing stable anatomical delineation across slice levels.

**Figure 8:**
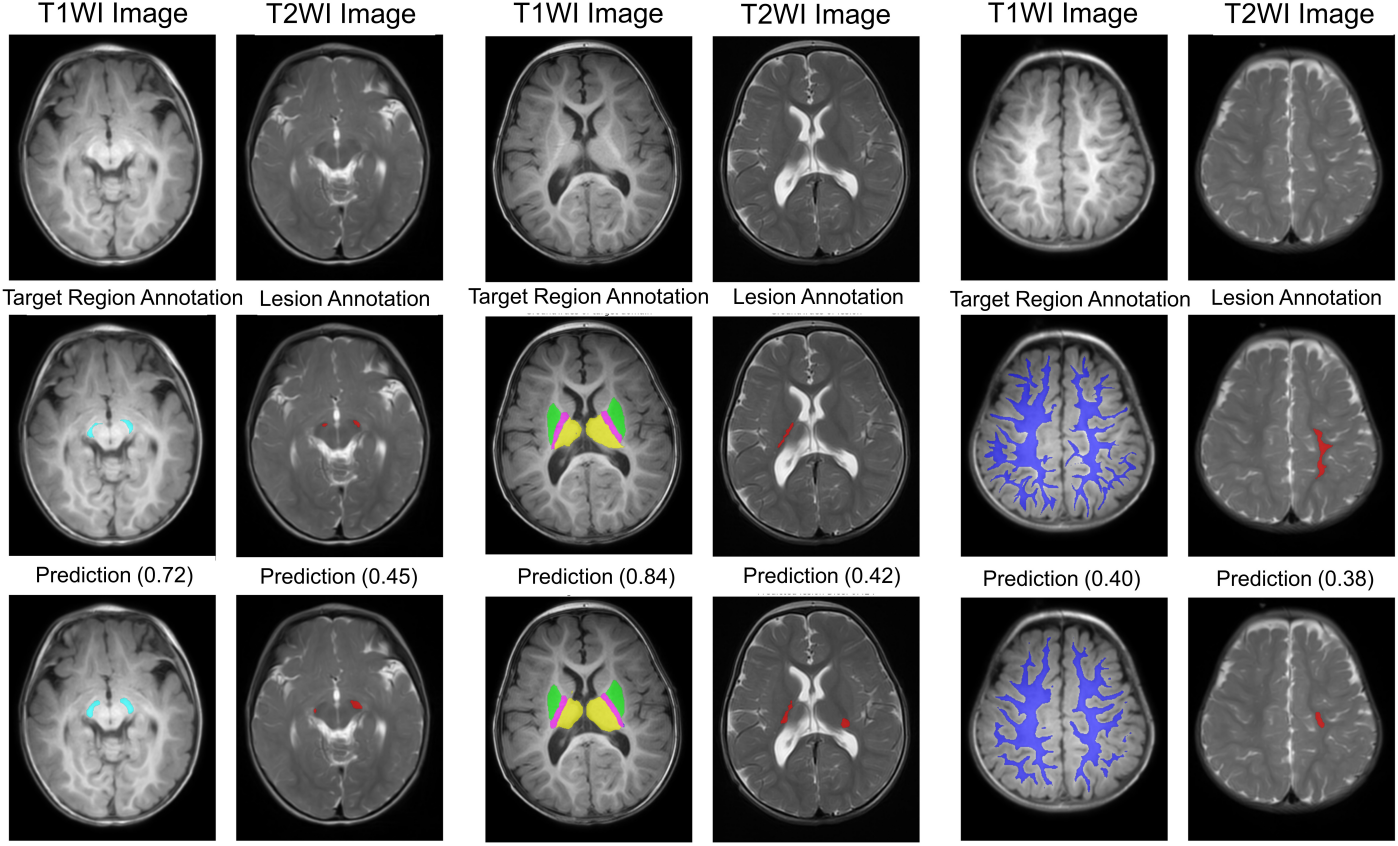
Representative MMSeg-CP predictions showing successful lesion recovery and residual failure modes in low-contrast regions.

Quantitative metrics were complemented by qualitative inspection across Figures 4–8. For target regions segmentation, MMSeg-CP more faithfully recovered the shape and continuity of structures, whereas several baseline models showed contour shrinkage, incomplete segmentation, blurred borders, or anatomically implausible asymmetry (Figure 4). At the lesion level, MMSeg-CP captured a larger fraction of the lesion extent and produced contours that better matched the reference masks even when lesions were small, fragmented, or weakly contrasted. Competing methods more often showed under segmentation, scattered false positives, or loss of continuity (Figure 5). Across additional examples (Figures 6–8), MMSeg-CP maintained good agreement with the reference annotations for both anatomical and lesion masks. In difficult cases with weak contrast or near poorly defined cortical-adjacent regions, the model occasionally missed small lesion fragments or slightly underestimate the full lesion extent.

### 3.3 *Post-hoc* analyses

As shown in Table 7, Dice was markedly lower for small lesions (0.246 ± 0.032) than for medium (0.477 ± 0.069) and large (0.471 ± 0.035) lesions, indicating that the modest overall Dice is driven primarily by the smallest lesions. An exploratory threshold scan identified a mean candidate threshold of 1,312 pixels, lesions at or below this threshold had substantially lower Dice than larger lesions (0.170 ± 0.072 vs. 0.454 ± 0.039, *P* = 0.006; Supplementary Table S5).

**Table 7:** Lesion-size stratified dice performance.

Lesion-size group	N	Mean n per fold	Dice (mean ±SD)	Median dice (mean ±SD)
Small	40	8.0	0.246 ± 0.032	0.259 ±0.079
Medium	37	7.4	0.477 ± 0.069	0.478 ±0.099
Large	40	8.0	0.471 ± 0.035	0.506 ±0.055

Independent of pixel-level overlap, a blinded clinician assessment of predicted lesion masks from 49 randomly selected cases achieved a localization success rate of 85.5% (42/49), with substantial inter-rater agreement (Cohen's κ = 0.74).

### 3.4 Subject-level aggregation analysis

The original any-positive-slice rule achieved high sensitivity (0.95 ± 0.05) but lower specificity (0.73 ± 0.08). Top-3 mean probability pooling achieved the best overall balance, with accuracy 0.88 ± 0.04, specificity 0.91 ± 0.05, and sensitivity 0.83 ± 0.10, reducing total false positives from 41 to 14 across the five folds, although false negatives increased from 5 to 15. Positive-slice-count thresholding (≥ 2 slices) showed a comparable trade-off (Supplementary Table S6).

### 3.5 Ablation study

At the overall task level, α = 0.5 yielded the highest lesion Dice and slice-level classification accuracy, while maintaining strong target-region Dice (Table 8).

**Table 8:** Overall segmentation and classification results under different loss-weight coefficients.

Weight coefficient	Target-region dice	Lesion dice	Slice accuracy	Subject accuracy	Subject specificity	Subject sensitivity
0.1	0.79	0.45	0.93	0.92	0.87	1.00
0.3	0.80	0.45	0.95	0.86	0.87	0.84
0.5	0.79	0.46	0.96	0.92	0.93	0.89
0.7	0.79	0.45	0.95	0.88	0.83	0.95

Region-wise Dice values varied only modestly across settings (Supplementary Table S7), with the most noticeable fluctuations in the centrum semiovale. The 0.5 setting also provided the best lesion HD95 (Supplementary Table S8).

Across subject-level metrics, α=0.5 achieved the best balance among accuracy, sensitivity, and specificity (Table 8). This configuration was selected as the default setting for MMSeg-CP.

## 4 Discussion

This study demonstrated that MMSeg-CP effectively integrates anatomical and pathological information from infant multimodal MRI through a unified multi-task framework. The proposed framework enhances the overall coordination among target-region segmentation, lesion delineation, and CP-related classification, rather than simply improving a single metric. MMSeg-CP achieved a mean target-region Dice of 0.79, lesion Dice of 0.41, a slice-level classification accuracy of 0.95, and a subject-level accuracy of 0.88, surpassing nine representative baselines in the overall balance of overlap, boundary, and diagnostic metrics. These results suggest that combining structural localization with pathology detection within a unified architecture not only enhances pixel-wise accuracy but also enhances case-level predictive reliability for early CP screening in infants with PVWMI.

### 4.1 Network architecture improvement

The MMSeg-CP model proposed in this study introduces enhancements across three dimensions: task design, network architecture, and training strategy. In contrast to traditional single-task models (Hassan et al., 2024), MMSeg-CP concurrently conducts target-region segmentation, intra-target lesion segmentation, and CP classification, thereby significantly improving feature utilization efficiency and cross-task synergy. The shared encoder enables simultaneous learning of minute lesion features and their clinical relevance, while the lightweight all-MLP decoder preserves spatial precision without excessive computational burden. Regarding the refined training strategy, ANTs-based two-dimensional affine registration ensures spatial alignment between T1WI and T2WI, with resolution standardized to 512 × 512 to eliminate inter-scanner variability. Data augmentation, loss function weighting, and exponential learning rate decay collectively mitigate sample imbalance and lesion sparsity. Furthermore, this end-to-end multi-task collaborative training paradigm effectively mitigates the error accumulation typically associated with traditional cascade models, facilitating direct mapping and optimization from imaging input to clinical diagnostic decision-making.

### 4.2 Segmentation performance

Compared with prior neonatal brain segmentation studies that focus on relatively well-defined structures (Chen et al., 2023), the five target regions in this study present greater challenges. Among the five target regions, the thalamus and lentiform nucleus achieved the highest Dice, due to their distinct boundaries and high gray-white matter contrast. Conversely, the PLIC yielded the lowest Dice of 0.72, likely resulting from its slender, elongated morphology and partial volume effects associated with slice thickness. The HD95 results demonstrated a similar trend: structures with simpler forms and sharper boundaries exhibited smaller boundary deviations. The centrum semiovale showed the poorest HD95, reflecting the network's remaining challenges in delineating complex regional boundaries. Overall, MMSeg-CP attained a mean Dice of 0.79 and HD95 of 3.06 across the five target regions, indicating it ability to maintain high anatomical discriminability despite the low-contrast characteristics of infant brain MRI, thereby meeting the requirements for lesion localization.

Compared with previous study based on single-modal T2WI for detecting diffuse white matter hyperintensities (Li et al., 2019), this study utilized multimodal imaging (T1WI + T2WI) to obtain richer image information and detect smaller lesions. MMSeg-CP achieved a mean Dice of 0.41 and HD95 of 11.91 for lesion segmentation. Although the performance in lesion segmentation was not as robust as that for target-region segmentation, these results must be considered in the context of the inherent characteristics of PVWMI lesions. PVWMI lesions are generally small and exhibit indistinct boundaries, with the smallest lesions comprising only a few dozen voxels. Additionally, the majority of images had a slice thickness of 5–6 mm, which caused tiny lesions to appear discontinuous or isolated across slices in the through-plane direction, thereby significantly complicating segmentation efforts. However, stratified analysis showed that the performance drop was concentrated in the smallest lesions (Dice 0.25), whereas medium and large lesions achieving acceptable overlap (Dice 0.47). Independent clinician assessment further confirmed that 87.5% of predicted masks achieved successful anatomical localization despite modest pixel-level overlap, suggesting that the model's segmentation output is clinically usable even when absolute Dice values are moderate.

### 4.3 Classification performance

Compared with previous studies confined to CP lesion detection (Simarro et al., 2025), MMSeg-CP advances beyond localization by enabling direct CP classification. The network achieved a slice-level classification accuracy of 0.95, demonstrating excellent discriminative capability between slices containing lesions and those with normal. At the subject level, CP classification accuracy reached 0.88, with a sensitivity of 0.90 and a specificity of 0.88, all outperforming the nine existing baseline networks. This integration yields balanced sensitivity and specificity, which is clinically relevant for minimizing missed diagnoses in high-risk infants while alleviating undue anxiety from false-positive referrals.

The subject-level aggregation analysis further clarifies the trade-off between sensitivity and specificity in 2D slice-wise modeling. The original any-positive-slice rule was intentionally sensitivity oriented and is appropriate for screening scenarios in which missed CP-associated lesions should be minimized. However, it can increase false positives when a single slice is affected by noise, artifacts, or overconfident prediction. Probability-based pooling (e.g., top-3 mean) provided a more balanced decision rule by substantially improving specificity while maintaining acceptable sensitivity. These findings suggest that aggregation strategies should be tailored to clinical context sensitivity prioritized rules for early triage, and conservative pooling for confirmatory decision support.

### 4.4 Ablation study analysis

The loss-weight coefficient α modulates the trade-off between anatomical supervision, lesion detection, and classification. At the overall task level, α = 0.5 yielded the best balance across lesion Dice, slice-level accuracy, and subject-level metrics (Table 8), whereas higher values (α = 0.7) marginally improved target-region boundary precision (HD95) at the expense of lesion specificity. Region-wise analysis showed that the anatomical branch remained robust to moderate changes in α, with the most pronounced fluctuations in structurally irregular regions such as the centrum semiovale (Supplementary Table S7). This relative stability suggests that the shared encoder preserves anatomical representations even when task weights vary, whereas the lesion and classification branches are more sensitive to optimization balance.

The clinical interpretation of this loss-weight coefficient depends on the intended use of the model. While sensitivity is critical for early CP screening, MMSeg-CP is designed as a decision-support tool rather than a standalone diagnostic replacement, excessive false positives therefore also carry tangible costs, such as unnecessary follow-up, family anxiety, additional workload. Thus, α = 0.5 was selected for its favorable balance across lesion localization, classification accuracy, sensitivity, and specificity.

In terms of clinical deployment, the proposed pipeline offers two practical advantages for early CP screening. First, the mean inference time of 51.7 ms per subject indicates that MMSeg-CP may be efficiently integrated into clinical Picture Archiving and Communication Systems or MRI review workflows, thereby providing real-time decision support for non-specialist clinicians. Second, by automating the detection of the five MRI predictors, the model reduces reliance on subjective radiological interpretation, which is particularly beneficial in centers with limited expertise in pediatric neuroradiology.

This study has several limitations. First, the study was retrospective and derived from a single institutional cohort with a modest sample size, so broader external validation is still needed before clinical deployment. We are currently organizing a multicenter cohort to validate the generalizability of MMSeg-CP in a subsequent study. Second, the lesion segmentation task remains difficult because many lesions are extremely small and poorly contrasted, which constrains the achievable Dice values even when qualitative localization is reasonable. Third, the present framework is based on 2D slice-wise analysis. Although this choice is justified by the coarse through-plane resolution of the data, future studies could investigate whether richer inter-slice aggregation strategies improve subject-level prediction. Fourth, baseline models were evaluated using their originally published configurations without extensive task-specific reoptimization, which may have underestimated their potential performance. Future multicenter studies should include systematic task-specific reoptimization of comparison models. Finally, the loss-weight coefficient was selected only through internal ablation analysis. Multicenter validation is therefore needed to evaluate whether scenario-specific α values are required.

## 5 Conclusion

This study presents MMSeg-CP, a multi-task SegFormer-based framework for early CP prediction in infants with PVWMI from multimodal MRI. The model achieved stable performance across segmentation and classification tasks, offering a favorable balance between overlap accuracy, boundary precision, and diagnostic discrimination. These findings support the continued development of anatomically informed AI systems for early CP-related MRI assessment and provide a practical foundation for future work on more robust and clinically transferable infant neuroimaging models.

## Data Availability

The raw data supporting the conclusions of this article will be made available by the authors, without undue reservation.
