# Supplementary material for: A multi-task segFormer framework for lesion segmentation and cerebral palsy classification based on multi-modal MRI in infant with periventricular white matter injury
Source: Front Neurosci. 2026 Jul 2;20:1870625. doi: 10.3389/fnins.2026.1870625 (PMC13372756; doi:10.3389/fnins.2026.1870625)
Supplement: Supplementary file 1 [file Data_Sheet_1.docx]

Supplementary Material

# Supplementary Tables

**METHODS**

**MRI Protocol**

MRI scans were obtained using 3.0T and 1.5T MRI scanners (Ingenia 3.0T or Achieva 1.5T, Philips Medical Systems, Best). The MRI equipment and imaging protocols are detailed in the Table S1. All infants received sedation or anesthesia according to clinical standard procedures during MRI examination.

# Table S1: The MRI machines and scanning protocols used at the five hospitals

| **Manufacturer** | **Scanner** | **Protocol** | **Scanning parameter** | | | | |
| --- | --- | --- | --- | --- | --- | --- | --- |
| Philips |  |  | TR  (ms) | TE  (ms) | Slice thickness  (mm) | FOV  (mm) | Matrix |
|  | Achieva  1.5T | T1WI | 406 | 15 | 7 | 230 | 224×159 |
|  |  | T2WI | 3136 | 100 | 7 | 230 | 252×171 |
|  |  | T2 FLAIR | 6000 | 120 | 7 | 230 | 208×144 |
|  |  | Sagittal T2WI | 1668 | 100 | 7 | 240 | 252×221 |
|  | Ingenia  3.0T | T1WI | 250 | 2.3 | 6 | 230 | 260×150 |
|  |  | T2WI | 1840 | 80 | 6 | 230 | 256×256 |
|  |  | T2 FLAIR | 7000 | 120 | 6 | 230 | 356×157 |
|  |  | Sagittal T2WI | 1946 | 80 | 6 | 230 | 308×238 |

FOV, field of view; MRI, Magnetic Resonance Imaging; T1WI, T1-weighted imaging; T2WI, T2-weighted imaging; T2-FLAIR, T2-fluid attenuated inversion recovery; TR, repetition time; TE, echo time.

**Table S2.** Summary of case numbers by slice number and thickness.

| **Slice number, Thickness** | **PVWMI with non** **-** **CP** | **PVWMI with CP** | **Normal cases** | **Total cases** |
| --- | --- | --- | --- | --- |
| 14, 6 mm | 0 | 1 | 0 | 1 |
| 15, 6 mm | 5 | 13 | 8 | 26 |
| 15, 7 mm | 4 | 0 | 0 | 4 |
| 18, 5 mm | 18 | 68 | 99 | 185 |
| 18, 6 mm | 5 | 8 | 14 | 27 |
| Total | 32 | 90 | 121 | 243 |

CP: cerebral palsy; PVWMI: periventricular white matter injury.

**Table S3.** Number of lesion-containing slices across CP cases.

| **Lesion-containing slices** | 2 | 3 | 4 | 5 | 6 | Total |
| --- | --- | --- | --- | --- | --- | --- |
| **Number of CP cases** | 2 | 11 | 58 | 18 | 1 | 90 |

CP: cerebral palsy.

**Table S4.** Inter-rater reliability of manual annotations.

| **Structure / Lesion** | **Dice (mean ± SD)** | **95% CI** | **Interpretation** |
| --- | --- | --- | --- |
| T1WI target regions |  |  |  |
| Thalamus | 0.87 ± 0.05 | 0.85, 0.89 | Excellent |
| Lentiform nucleus | 0.84 ± 0.06 | 0.72, 0.86 | Excellent |
| Cerebral peduncle | 0.79 ± 0.07 | 0.67, 0.81 | Good |
| PLIC | 0.71 ± 0.09 | 0.58, 0.74 | Good |
| Centrum semiovale | 0.67 ± 0.11 | 0.54, 0.70 | Moderate |
| Mean across target regions | 0.78 ± 0.06 | 0.76, 0.80 | Good |
| T2WI lesions (overall) | 0.64 ± 0.13 | 0.40, 0.68 | Moderate |
| Slice-level CP classification |  |  |  |
| Cohen’s kappa | 0.81 | 0.75, 0.87 | Substantial agreement |

PLIC, posterior limb of the internal capsule; CI, confidence interval.

**Table S5**. Exploratory lesion-burden threshold analysis.

| **Fold** | **Candidate threshold** | **n≤ threshold** | **n> threshold** | **Dice≤ threshold** | **Dice> threshold** | **Difference** | ***P* value** |
| --- | --- | --- | --- | --- | --- | --- | --- |
| 1 | 1518 | 3 | 17 | 0.138 | 0.442 | 0.304 | 0.019 |
| 2 | 1039 | 6 | 20 | 0.181 | 0.464 | 0.283 | 0.001 |
| 3 | 1614 | 4 | 16 | 0.232 | 0.489 | 0.257 | 0.011 |
| 4 | 645 | 5 | 21 | 0.064 | 0.393 | 0.329 | ＜0.001 |
| 5 | 1746 | 7 | 18 | 0.236 | 0.484 | 0.248 | ＜0.001 |
| Mean ± SD | 1312.4±458.6 | — | — | 0.170±0.072 | 0.454±0.039 | 0.284±0.033 | 0.006 |

**Table S6**. Subject-level aggregation strategies for CP classification.

| **Aggregation strategy** | **Definition** | **Accuracy** | **Specificity** | **Sensitivity** |
| --- | --- | --- | --- | --- |
| Any positive slice | Subject is CP-positive if any slice is predicted CP-positive | 0.810± 0.046 | 0.729 ± 0.081 | 0.945 ± 0.053 |
| Max probability pooling | Maximum slice-level CP probability within a subject | 0.810 ± 0.046 | 0.729 ± 0.081 | 0.945 ± 0.053 |
| Noisy-or pooling | $1-\prod_{i} (1-p_{i})$, where pi is slice-level CP probability | 0.810 ± 0.046 | 0.729± 0.081 | 0.945 ± 0.053 |
| Top-2 mean probability pooling | Mean of the two highest slice-level CP probabilities | 0.860± 0.048 | 0.846 ± 0.063 | 0.876± 0.080 |
| Positive slice count ≥ 2 | Subject is CP-positive if at least two slices are predicted CP-positive | 0.877 ± 0.047 | 0.900 ± 0.049 | 0.828 ± 0.099 |
| Top-3 mean probability pooling | Mean of the three highest slice-level CP probabilities | 0.881 ± 0.045 | 0.906 ± 0.047 | 0.828 ± 0.099 |
| Top-4 mean probability pooling | Mean of the four highest slice-level CP probabilities | 0.864± 0.043 | 0.939 ± 0.032 | 0.727 ± 0.137 |
| Positive slice count ≥ 3 | Subject is CP-positive if at least three slices are predicted CP-positive | 0.815 ± 0.040 | 0.952 ± 0.041 | 0.574 ± 0.092 |
| Positive slice count ≥ 4 | Subject is CP-positive if at least four slices are predicted CP-positive | 0.798 ± 0.034 | 1.000 ± 0.000 | 0.452 ± 0.095 |
| Mean probability pooling | Mean slice-level CP probability across all slices | 0.629 ± 0.043 | 1.000 ± 0.000 | 0.000 ± 0.000 |

**Table S7.** Region-wise Dice under different loss-weight coefficients.

| **Weight**  **Coefficient** | **Centrum**  **semiovale** | **PLIC** | **Cerebral**  **peduncle** | **Thalamus** | **Lentiform**  **nucleus** | **Mean** |
| --- | --- | --- | --- | --- | --- | --- |
| 0.1 | 0.74 | 0.73 | 0.73 | 0.91 | 0.86 | 0.79 |
| 0.3 | 0.76 | 0.74 | 0.74 | 0.90 | 0.86 | 0.80 |
| 0.5 | 0.73 | 0.74 | 0.74 | 0.89 | 0.87 | 0.79 |
| 0.7 | 0.73 | 0.73 | 0.75 | 0.89 | 0.86 | 0.79 |

**Table S8.** Region-wise HD95 and lesion HD95 under different loss-weight coefficients.

| **Weight**  **coefficient** | **Centrum**  **semiovale** | **PLIC** | **Cerebral**  **peduncle** | **Thalamus** | **Lentiform**  **nucleus** | **Mean** | **Lesion**  **HD95** |
| --- | --- | --- | --- | --- | --- | --- | --- |
| 0.1 | 8.49 | 2.22 | 1.67 | 2.24 | 3.19 | 3.56 | 12.44 |
| 0.3 | 8.30 | 2.18 | 1.53 | 2.20 | 3.49 | 3.54 | 12.49 |
| 0.5 | 8.44 | 2.14 | 1.56 | 2.59 | 3.38 | 3.62 | 11.19 |
| 0.7 | 8.51 | 1.93 | 1.56 | 2.14 | 2.78 | 3.38 | 11.42 |

# Supplementary Figures 1

##
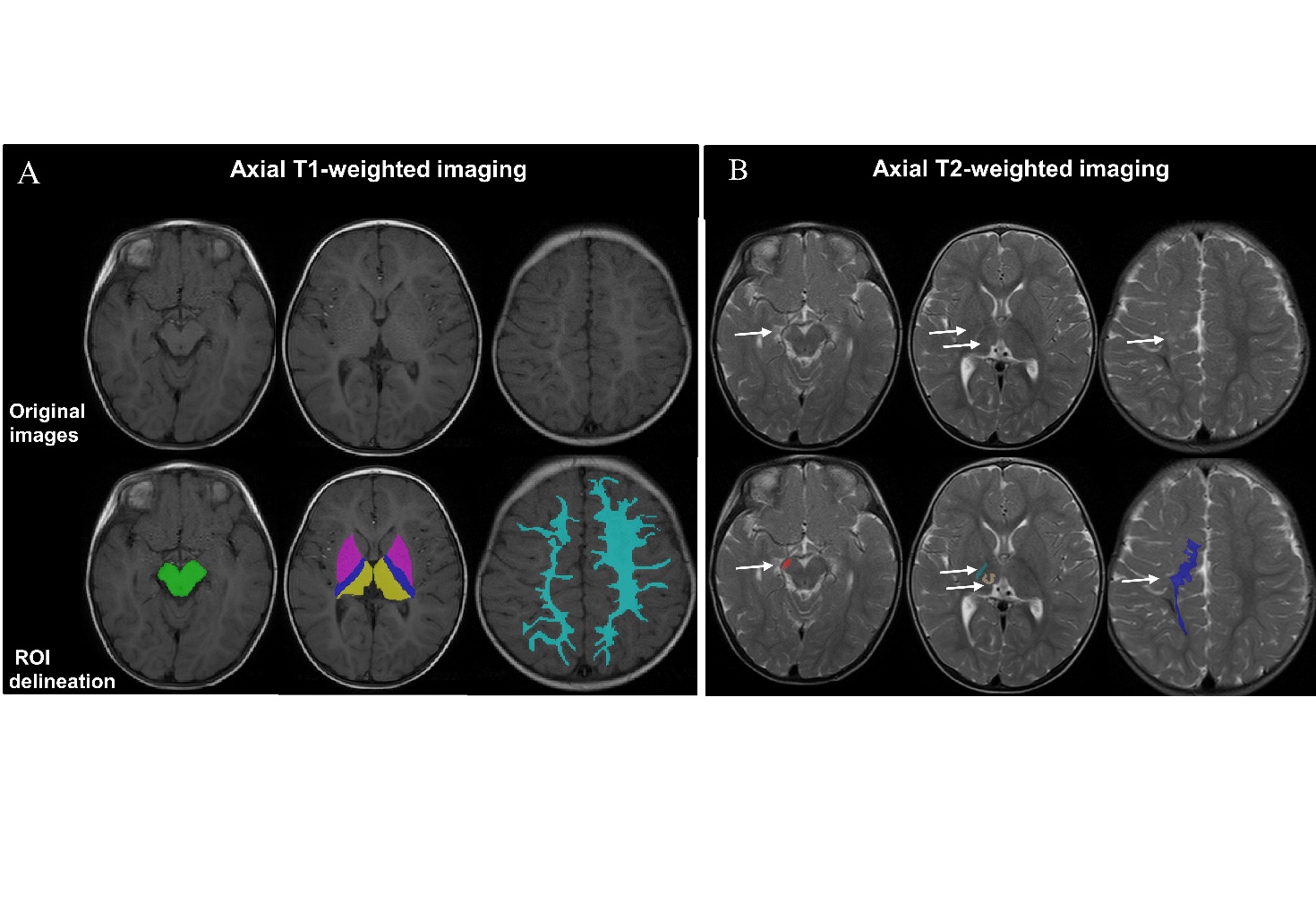


Figure 1. Schematic delineation of the five anatomical target regions and their corresponding lesions. (A) Axial T1WI showing the five key anatomical target regions (first row) and the corresponding manually delineated ROIs (second row). Cerebral peduncle (green), PLIC (dark blue), thalamus (yellow), lentiform nucleus (rose red), and centrum semiovale (light blue). (B) Axial T2WI showing the lesions within the five target regions (first row) and the corresponding lesion delineated ROIs (second row, arrows).

**
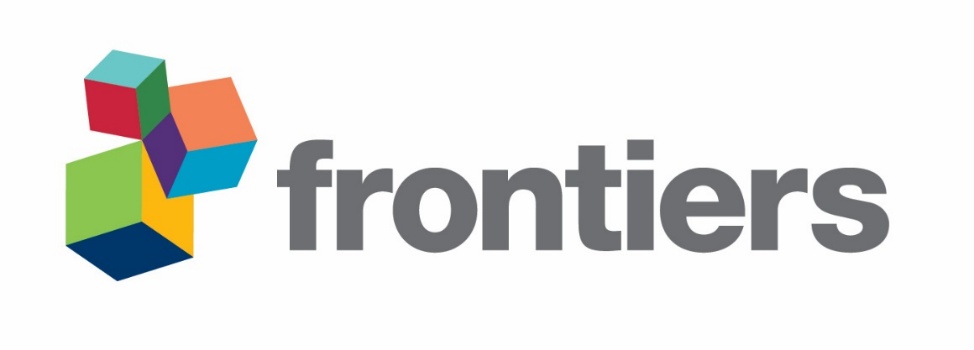
**
